# Neutralizing and Epitope-Specific Antibodies against Respiratory Syncytial Virus in Maternal and Cord Blood Paired Samples

**DOI:** 10.3390/v14122702

**Published:** 2022-12-02

**Authors:** Fumi Mashiyama, Koichi Hashimoto, Sakurako Norito, Hisao Okabe, Akiko Sato, Yohei Kume, Ryo Maeda, Maki Sato, Masatoki Sato, Hyo Kyozuka, Keiya Fujimori, Hidekazu Nishigori, Kosei Shinoki, Seiji Yasumura, Hiroko Sakuma, Mitsuaki Hosoya

**Affiliations:** 1 Department of Pediatrics, School of Medicine, Fukushima Medical University, Fukushima 960-1295, Japan; 2 Pediatrics, Hoshi General Hospital, Koriyama 960-1295, Japan; 3 Fukushima Regional Center for the Japan Environmental and Children’s Study, Fukushima 960-1295, Japan; 4 Department of Obstetrics and Gynecology, School of Medicine, Fukushima Medical University, Fukushima 960-1295, Japan; 5 Fukushima Medical Center for Children and Women, Fukushima Medical University, Fukushima 960-1295, Japan; 6 Department of Public Health, School of Medicine, Fukushima Medical University, Fukushima 960-1295, Japan

**Keywords:** respiratory syncytial virus, neutralizing antibody, epitope-specific antibody, trans-placental antibody, site zero, cord blood

## Abstract

Only a few qualitative studies of neutralizing antibody titers (NATs) against respiratory syncytial virus (RSV) have focused on epitope-specific antibody (ESA) levels. Here, NATs against RSV in sera were measured using the blood of 412 mothers and cord blood (CB) of 95 of the 412 mother–child pairs. ESA levels against sites zero (Ø) and IIa of the F protein of RSV were measured in 87 of the 95 mother–child pairs. The median gestational age was 39 weeks. The NATs and ESA levels in CB were slightly higher than those in maternal blood (MB). The NATs for RSV subtype A (RSV-A) in MB and CB showed a positive correlation (r = 0.75). The ESA levels against sites Ø and IIa in MB and CB showed positive correlations, r = 0.76 and r = 0.69, respectively. In MB, the NATs and ESA levels against RSV were positively correlated, more significantly against site Ø (RSV-A: r = 0.70, RSV-B: r = 0.48) than against site IIa (RSV-A: r = 0.19, RSV-B: r = 0.31). Sufficient amounts of ESAs against sites Ø and IIa of RSV were transferred from mothers to term infants. ESA levels against site Ø contribute to NATs.

## 1. Introduction

Respiratory syncytial virus (RSV) causes lower respiratory tract infections and acute respiratory failure in infants. RSV infection is the second-leading cause of infectious disease-related deaths worldwide after malaria, and approximately half of all deaths due to lower respiratory tract infections during the neonatal period (less than 28 days of age) are caused by RSV infection [1]. In developed countries, including Japan, most infants are infected with RSV by the age of 3 years, and 2–10% of all infants hospitalized have severe RSV infection [1]. Palivizumab (Synagis^®^), a monoclonal antibody against the RSV fusion F protein, is the only drug for RSV infection and is indicated only in high-risk pediatric patients for the prevention of severe RSV infection [2]. However, no effective treatment or universal prophylaxis is available for RSV infection, and the World Health Organization designates RSV infection as an infectious disease that requires immediate action [3].

**Figure 1:**
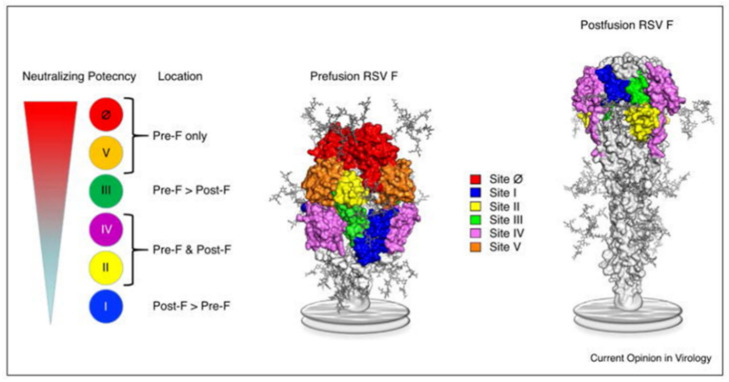
Location of the major antigenic sites on pre-F and post-F conformations of RSV. This figure is adopted from Graham [6] (The copyright has been obtained from the corresponding author).

RSV (family Pneumoviridae) is a negative-sense single-stranded, enveloped RNA virus. The RSV particle envelope contains G, F, and SH spike proteins associated with neutralizing activity. RSV has a single serotype with two antigenic subtypes, subtype A (RSV-A) and subtype B (RSV-B). The amino acid sequence of the G protein varies by approximately 50% between RSV-A and RSV-B. RSV is also classified into RSV-A and RSV-B based on the G protein gene sequence, but there are other genotypes associated with the annual prevalence of RSV. However, the involvement of subtype and genotype in the severity of RSV infection is controversial. Of the envelope proteins, only the F protein is essential for cell infection; the epitope sequence of the F protein is relatively conserved [4,5]. The F protein can be defined as a structurally metastable prefusion type (pre-F) or stable postfusion type (post-F). Six epitopes (sites I, II, III, IV, V, and Ø [zero]) in the F protein have been reported, and epitope-specific antibodies (ESAs) have been developed [6,7,8] (Figure 1) [6]. Site II exists in both pre-F and post-F and contains subtypes a and b. Subtype a (IIa) is the antigen-recognition site of palivizumab [2]. Among these epitopes, site Ø existing in pre-F, which induces a high-titer neutralizing antibody (NA), has garnered attention [9,10]. The neutralizing activity of monoclonal antibodies against site Ø is 50–100 fold higher than that of palivizumab [11,12], and the use of one of these antibodies, nirsevimab, considerably reduces the consultation and hospitalization rates for RSV-related lower respiratory tract infections in healthy, early-term infants under 1 year of age [13]. In RSV serum epidemiology, the primary method for assessing RSV immunity is the measurement of NAs; it quantifies the serum functional capacity to neutralize RSV infectivity [14]. Infants born to mothers with high NA titers (NATs) against RSV are spared from severe RSV infections during the first few months of life [15], and those with high NATs against RSV do not experience severe RSV infections [16]. This fact shaped the current RSV prophylaxis, which involves the administration of anti-RSV monoclonal antibody, palivizumab, to high-risk children. Several vaccines against RSV have been developed, and ongoing clinical trials are testing a live attenuated vaccine for infants and a subunit vaccine (maternal vaccine) for pregnant women [17]. The transfer of maternal antibodies to infants has been investigated using NATs against live RSV and the titer of a palivizumab-like antibody against site IIa [18]. However, only a few qualitative evaluations of NAs against RSVs in neonates have been conducted that have considered specific antibodies against each neutralizing epitope, including site Ø [19,20]. Hence, here, we measured NATs against RSV and neutralizing ESA levels using mother–infant paired serum samples collected at birth to qualitatively evaluate the NAs against RSV in transition antibodies.

## 2. Materials and Methods

### 2.1. Participants

**Figure 2:**
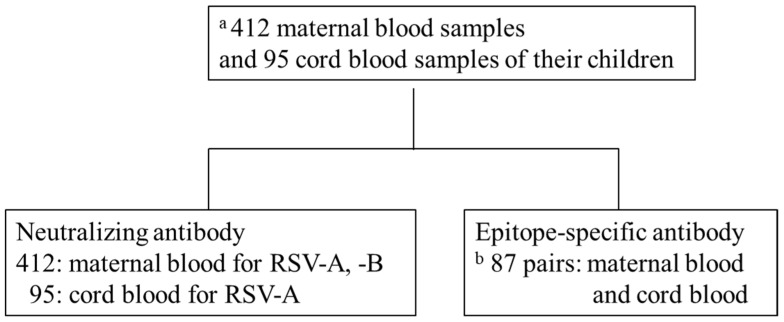
Schematic diagram of analysis. (a) Of 9,643 births in Fukushima Prefecture from November 2011 to April 2014, 412 maternal blood (MB) and 95 cord blood (CB) samples of the offspring were available for analysis. (b) Eighty-seven pairs of 97 samples in which neutralizing antibodies were measured.

This was an adjunct study of the Japan Environment and Children’s Study (JECS) by the Fukushima Regional Center, a nationwide prospective birth cohort study [21,22]. We measured NATs in the blood samples of 412 mothers participating in the JECS who gave birth in Fukushima Prefecture between November 2011 and April 2014. Furthermore, 95 paired cord blood (CB) samples were analyzed for NATs and ESA levels simultaneously; eight samples had measurement errors. Hence, ESA level was measured in 87 paired samples (Figure 2). NATs and ESA levels were measured using only samples of sufficient volume for analysis. Information about this study was provided on our website and via a newsletter mailed to all participants of the JECS. Mothers who did not agree to participate were excluded from the study (opt-out system). This study was approved by the ethics committee of the Ministry of the Environment and Fukushima Medical University (number #2124). Perinatal and postnatal information was obtained from JECS dataset jecs-ag-20180131. Maternal background information included maternal age, gestational age, delivery type, and number of siblings living together. Neonatal information included birth weight, sex, premature birth, and umbilical artery blood pH.

### 2.2. Viruses and Cells

For RSV-A and RSV-B, the experimental standard strain A2 (American Tissue Culture Collection [ATCC], Rockville, MD, USA) and clinical isolate of genotype GB3 were used, respectively. RSV was proliferated in HEp-2 cells (ATCC CCL-23) and virus stocks were stored at −80 °C until use.

### 2.3. Cell Culture

Minimum essential medium (MEM) (Thermo Fisher Scientific, Waltham, MA, USA) containing 10% heat-inactivated fetal bovine serum (FBS) (Thermo Fisher Scientific) was used. The growth medium (GM) was 10% FBS-MEM supplemented with L-glutamine (30 mg/mL), penicillin (50,000 U/mL), gentamicin (50 µg/mL), and fungizone (5 mg/mL). HEp-2 cells were cultured in GM at 37 °C in a 5% CO_2_ incubator. All reagents for GM supplementation, except FBS, were obtained from FUJIFILM Wako Pure Chemical Corporation (Osaka, Japan).

### 2.4. Virus Titration

The virus was serially diluted using maintenance medium (MM), 5% FBS-MEM. The diluted virus solution was inoculated into HEp-2 cells cultured to a monolayer in 96-well microplates, which were then incubated in MM for 5 days. The cytopathic effects (CPE) were observed under a microscope at eight wells per virus dilution, and the virus titer was determined from the 50% tissue culture infectious dose (TCID_50_).

### 2.5. Neutralization Test

Serum samples were inactivated at 56 °C for 30 min and diluted in two-fold steps from 1:4 to 1:512 using MM. Thereafter, 200 TCID_50_/0.05 mL virus was added to each well of a 96-well plate with 0.05 mL of diluted serum, followed by incubation for 2 h at 37 °C in an incubator with 5% CO_2_. One hundred microliters of the mixture of virus and serum was added to each well of a 96-well microplate containing HEp-2 cell monolayer, followed by incubation for 5 days at 34 °C in an incubator supplemented with 5% CO_2_; the CPE was observed under an optical microscope. NAT was determined as the highest serum dilution that inhibits the CPE by 50%; each measurement was conducted in duplicate.

### 2.6. Determination of ESA Levels against RSV

ESA level was measured using a direct competitive enzyme-linked immunosorbent assay. Neutralizing epitopes were sites Ø and IIa, which is a selective epitope of palivizumab. Antibody titers were measured according to the method reported by Jounai et al. [23]. The pre-F protein antigen and site Ø-recognizing antibody (AM22) were provided by Daiichi Sankyo Co., Ltd., Tokyo, Japan, under a Material Transfer Agreement contract. Using maternal blood (MB) and CB serum, the competitive binding inhibition rate with site Ø recognition antibody (AM22) and site IIa recognition antibody (Synagis^®^) against pre-F protein was calculated. A standard curve was created from the inhibition rate plot of each serum dilution step using JMP^®^ 14.0.0 (SAS Institute Inc., Cary, NC, USA), and each antibody concentration in serum was calculated. The lower limit of detection (LLoD) was 20 μg/mL for both sites II and Ø. Samples with a concentration below the LLoD were assigned a value of 10 μg/mL.

### 2.7. Statistical Analysis

Statistical analysis was conducted using BellCurve for Excel (Social Survey Research Information Co., Ltd., Tokyo, Japan). The corresponding continuous variables were compared using a paired *t*-test, and non-corresponding continuous variables were compared using Mann–Whitney test. Pearson’s product-moment correlation coefficient was used to determine the correlation of continuous variables. Kruskal–Wallis test was used for comparing NATs and ESA levels between the groups. Binary variables were analyzed using the chi-square test and Fisher’s exact probability test if the minimum expected frequency was less than 5. Results with *p* < 0.05 were considered statistically significant. Cohen’s d was used to evaluate effect size.

## 3. Results

### 3.1. Maternal and Infant Background

The NATs of the mothers and newborns were measured in 412 MB and 95 CB samples, respectively. The median age of the 412 mothers was 31 (interquartile range [IQR]: 27–34) years. The median gestational age was 39 weeks and 3 days (IQR: 38 weeks 2 days to 40 weeks 2 days). Furthermore, 227 mothers (55.2%) lived with the newborn’s sibling. The median birth weight was 2991 (IQR: 2733–3253) g, and 208 newborns were boys (50.5%). There was no difference in backgrounds between the overall 412 pairs and 95 pairs in which NAs could be analyzed (Table 1).

### 3.2. NATs against RSV-A and -B in MB

**Figure 3:**
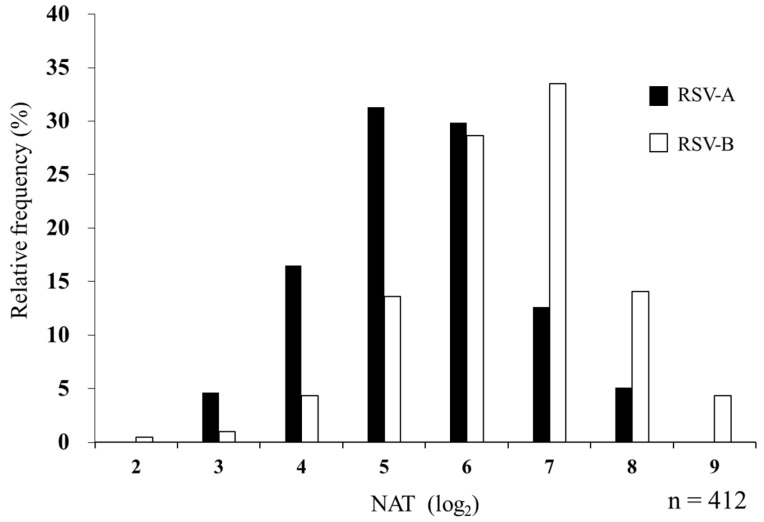
Relative frequency histogram of serum NAT against RSV-A and RSV-B in maternal blood. In total, 412 MB samples were evaluated for NATs against RSV-A and RSV-B. The maximum dilution of serum that suppressed the appearance of viral CPE by 50% is represented by log_2_ as NAT. Black and white bars indicate NATs against RSV-A and RSV-B, respectively.

The median NAT against RSV was 1:32 for subtype A and 1:64 for subtype B in MB. The geometric mean titers (95% confidence interval [CI]) were 5.45 (5.33–5.56) for RSV-A and 6.48 (6.36–6.60) for RSV-B. Subtype B showed a higher titer (*p* < 0.001, Cohen’s d = 0.85) (Table 2). The distribution of log_2_ serum NATs is shown in Figure 3. Subtypes A and B showed a positive correlation (r = 0.54, *p* < 0.001), and the kappa coefficient of 0.40 suggested a moderate agreement.

### 3.3. NATs against RSV-A in MB and CB

The NATs against RSV-A in MB and CB were compared among the 95 pairs that could be analyzed simultaneously (Figure 2). The median NAT against RSV-A was 1:32 for MB and 1:64 for CB. The geometric mean titers (95% CI) were 5.49 (5.25–5.73) and 5.91 (5.69–6.12) for MB and CB, respectively (*p* < 0.0103, d = 0.36) (Table 3). These titers also showed a strong positive correlation (r = 0.75, *p* < 0.001); the kappa coefficient of 0.71 indicated a moderate agreement.

### 3.4. Amount of Anti-RSV-Specific Antibodies against Various Epitopes in MB and CB

**Figure 4:**
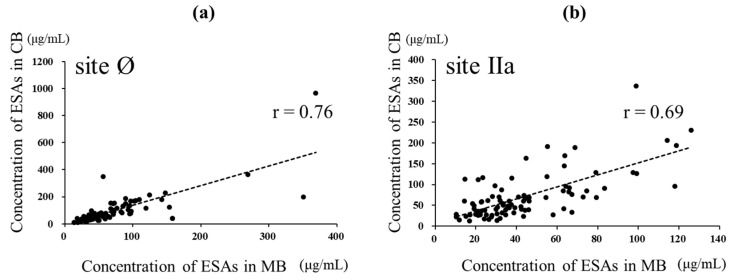
Relationship between ESAs specific to sites Ø and IIa in maternal and cord blood. In total, 87 MB and CB paired samples were evaluated for ESAs. (**a**) Relationship between the ESA level specific to site Ø in MB and CB. The broken line represents linear regression (r = 0.76, *p* < 0.001)). (**b**) Relationship between the level of ESA specific to site IIa in MB and CB. The broken line represents linear regression (r = 0.69, *p* < 0.001). Pearson’s product-moment correlation coefficient.

The anti-RSV ESA levels could be measured in 87 paired samples of the 95 paired samples of MB and CB in which NATs could be assessed simultaneously (Figure 2). The ESA level against site Ø in CB was higher than that in MB (*p* = 0.0092, d = 0.25), with median (IQR) levels of 64.97 (39.67–112.83) and 53.6 (36.27–85.57) μg/mL, respectively. The ESA levels against site Ø showed a strong positive correlation with both MB and CB (r = 0.76, *p* < 0.001). The ESA levels against site IIa in CB were higher than those in MB (p < 0.001, d = 0.63, with median (IQR) levels of 57.83 (33.21–89.05) and 36.43 (24.88–60.57) μg /mL, respectively. The levels of specific antibodies to site IIa also showed a strong positive correlation with both MB and CB (r = 0.69, p < 0.001) (Figure 4).

### 3.5. NATs and ESA Levels against RSV-A and RSV-B in MB

**Figure 5:**
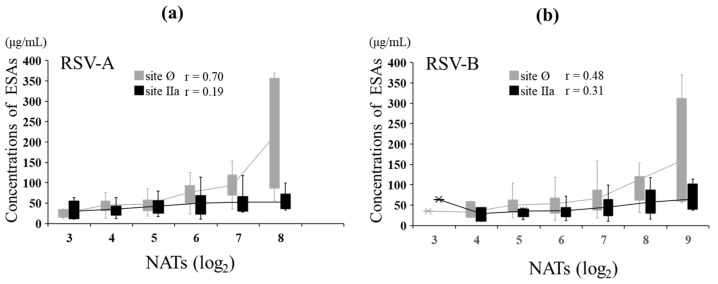
Relationship between NATs and ESAs in maternal blood. In total, 87 MB samples were evaluated for ESAs. The graph is shown as a box plot, showing the range of the 1st and 3rd quartiles as well as the maximum and minimum values. The polygonal line represents the average ESA levels at each NAT. (**a**) Relationship between the levels of ESAs specific to sites Ø and IIa and NATs against RSV type A in MB. (**b**) Relationship between the levels of ESA specific to sites Ø and IIa and NATs against RSV type B in MB. Pearson’s product-moment correlation.

In MB, the relationship between NATs and ESA levels against RSV-A was evaluated. The NATs against RSV-A showed a strong positive correlation with ESA levels to site Ø (r = 0.70, *p* < 0.001) and no correlation with ESA levels to site IIa (r = 0.19, *p* = 0.074). The NATs against RSV-B showed a moderate positive correlation with ESA levels to sites Ø (r = 0.48, *p* < 0.001) and IIa (r = 0.31, *p* = 0.004) (Figure 5). The level of ESAs to each epitope at the same NAT of both types was comparable between RSV-A and RSV-B against site IIa, but the level for RSV-A was more than that for RSV-B at high NATs against site Ø (Table 4).

## 4. Discussion

In this study, we qualitatively assessed NAs against RSV from transfer antibodies in pairs of mother and child sera at birth by measuring specific antibodies to neutralizing epitopes, sites Ø and IIa. We showed that a sufficient titer of ESAs against sites Ø and IIa of RSV is transferred from mothers to term infants; furthermore, ESAs against site Ø contribute to NATs.

In viral diseases, the protective effect of antibodies maternally transferred to the infant is well-known [24]. Antibodies induced by maternal vaccination are transferred to infants through the placenta [25]. Severe RSV infection in neonates is associated with low NATs, and low maternal transfer antibodies are a risk factor for severe RSV infection [15,16,18]. RSV is a recurrent infection, and most pregnant women have antibodies against RSV at delivery [26]. Generally, antibody transfer from the mother to the infant begins at approximately 13 weeks of gestation [27], reaches 50% of the maternal concentration at 28–32 weeks, shows a linear increase until 35 weeks with a slower increase thereafter [26], and ceases in the last 4 weeks. The serum IgG levels in term-born infants are the same as or higher than those in their mothers [28,29]. In agreement with the findings of Chu et al. [18], we observed a positive correlation between the NATs of MB and those of CB samples against RSV in full-term births, with CB tending to have higher titers. Similarly, the ESA levels correlated between MB and CB but were higher in CB. Therefore, ESAs and neutralizing activity are transferred and maintained between MB and CB in term-born infants. Koivistro et al. showed that antibodies against RSV-neutralizing epitope site Ø (pre-F antibodies), which are transfer antibodies from the mother, are fundamental for providing immune protection against RSV infection in the infant [30]. A report suggesting the effectiveness of maternal vaccines with RSV F protein nanoparticles [17] and the findings of this study substantiate the transfer of antibodies induced by maternal vaccines to infants, supporting the policy of introducing such vaccines.

High titers of RSV-NAs transferred from the mother prevent early RSV infection and aggravation. However, the antibody threshold for avoiding severe infection remains unclear. Piedra et al. reported the avoidance of acute respiratory infections caused by RSV with naturally acquired serum NAT [31]. Among 157 study participants, including 27 infants, those with an NAT of 6.0 log_2_ (arithmetic titer of 1/64) for RSV-A were 3.5-fold more likely to not have an RSV-related hospitalization than those with a titer of less than 6.0 log_2_. Similarly, those with an NAT of 8.0 log_2_ or higher (arithmetic antibody titer of 1/256) against RSV-B were 2.9-fold more likely to not have an RSV-related hospitalization than those with a titer < 8.0 log_2_. A clinical trial of palivizumab used to prevent severe disease in preterm infants showed that 40 μg/mL serum palivizumab is equivalent to 7.3 log_2_ of neutralizing titer and reduces hospitalization associated with RSV infection by 55% [32]. Chu et al. [18] showed that a reduced risk of lower respiratory tract infections is associated with higher CB neutralizing titers of 8.0 log_2_ or higher. However, these did not distinguish between RSV subgroups. Maternal NAT is influenced by the RSV strain used for NAT measurement or the epidemic situation of RSV infection. Here, 47.6% and 18.5% of mothers had an NAT of 6.0 log_2_ or higher for type A and 8.0 log_2_ or higher for type B, respectively, and 14.3% of the mothers met these two criteria simultaneously.

The amount of specific antibody against site Ø contributes to the high NATs against RSV. Adsorption treatment of human serum with pre-F protein reportedly reduces the RSV-neutralizing activity by more than 90%, whereas post-F protein reduces it by only approximately 30% [19]. In the protein competitive neutralization assay using pre-F protein mutants in which site Ø or II was modified to knock out antibody binding to the corresponding sites, these sites accounted for less than 35% and 10% neutralizing activity, respectively. In a binding competition assay using monoclonal antibodies, the amount of site Ø-specific antibody correlated with neutralization activity, but the magnitude of binding competition by the level of site II-specific antibody did not correlate with the neutralization activity. Antibodies recognizing site Ø showed the highest correlation with virus neutralization, but high levels of antibodies targeting other sites of the F protein could mediate a strong antiviral antibody response [20]. Here, site Ø was strongly correlated with both RSV-A and type B NATs, and the correlation with site IIa was weak.

Here, for high NATs such as 6.0 log_2_ and 7.0 log_2_, subtype B had lower ESA levels against site Ø than subtype A, even with the same NAT level, indicating that subtype B induces higher NATs with a lower ESA level. In addition, a positive correlation was observed between NATs and ESAs against site Ø in RSV-A and RSV-B. In particular, the positive correlation was stronger in RSV-A than in RSV-B. The site Ø-recognizing antibody (AM22) used in this study has a 50-fold higher affinity for subtype A than for subtype B, which is due to the amino acid differences in epitope Ø recognized by AM22 between subgroup A and B, which are Lys209 and Gln209, respectively [33]. These findings suggest that subtype-specific nAbs may recognize different amino acids within site Ø.

This study had limitations. First, in examining ESAs to site Ø, we did not use RSV-B high-affinity monoclonal antibodies such as RSD5-GL, which has a 2000-fold stronger affinity for RSV-B than for RSV-A, along with AM22 antibodies, which have a high affinity for subtype A [33]. The use of antibodies with high affinity for each subtype would have further clarified the association between NA activity and ESAs. Second, because CB samples at birth were not sufficient, not all analyses could be performed with paired MB and CB samples. Third, the study mostly included full-term mother–infant pairs, and the associations between preterm birth and NATs or ESA levels in CB samples were not determined. In general, insufficient IgG transfer occurs before 36 weeks of gestation, which is an indication for palivizumab administration to reduce RSV severity. Further studies including more preterm cases are needed. Fourth, because of the seasonality of RSV epidemics, it is necessary to consider the effect of the timing of specimen collection on the NA prevalence [34]. In recent years, the RSV epidemic season in Japan has changed from autumn/winter to summer [35], and the prevalent strains, including genotypes, also change with each epidemic season [36]; thus, epidemiological studies using clinical isolates over several seasons are warranted. Finally, because of the ambiguity of the children’s RSV infection status, we were unable to analyze the relationship between RSV infection and the number of NAs.

## 5. Conclusions

The titer of epitope-specific NAs against sites IIa and Ø of RSV in full-term infants was slightly higher than that in their mothers, and specific antibodies against site Ø contribute to NATs. We believe that qualitative analyses of maternally transferred epitope-specific NAs using maternal and infant paired samples at birth are valuable for the development of vaccines and monoclonal antibodies targeting site Ø.

## Figures and Tables

**Table 1:** Participant characteristics.

Characteristic	412 pairs	95 pairs	*p*
Maternal			
Age (years), median (IQR)	31 (27–34)	30 (27–33)	0.575
Gestational age (weeks (w) +days (d)), median (IQR)	39 w + 3 d (38 w + 2 d − 40 w + 2 d)	39 w + 4 d (38 w + 5 d − 40 w + 4 d)	0.137
Delivery type: cesarean section, n (%)	84 (20.4)	18 (19.0)	0.796
Children living together, n (%)	227 (55.2)	50 (52.6)	0.803
Neonates			
Birth weight (g), median (IQR)	2991 (2733–3253)	2992 (2720–3248)	0.594
Male, n (%)	208 (50.5)	53 (55.8)	0.602
Premature birth, n (%)	22 (5.3)	4 (4.2)	0.801
Umbilical artery blood pH, median (IQR)	7.33 (7.29–7.36)	7.32 (7.28–7.35)	0.443

The backgrounds of all 412 pairs of mothers and children, 95 of which had cord blood (CB) available, were compared. Continuous variables were analyzed using the *t*-test and binary variables with the chi-square test (Fisher’s exact probability test if the minimum expected frequency was less than 5).

**Table 2:** NATs against RSV-A and RSV-B in maternal blood.

Sample (n)	RSV-A (412)	RSV-B (412)	*p* * Cohen’s d
NAT, median (IQR)	1:32 (1:32–1:64)	1:64 (1:64–1:128)	-
GMT (95% CI)	5.45 (5.33–5.56)	6.48 (6.36–6.60)	<0.001 0.85

CI: confidence interval, GMT: geometric mean titer, IQR: interquartile range, NAT: neutralizing antibody titer, RSV: respiratory syncytial virus. The GMT is shown in log_2_. * Paired *t*-test.

**Table 3:** NATs against RSV type A in maternal and cord blood.

Sample (n)	Maternal Blood (95)	Cord Blood (95)	*p* * Cohen’s d
NAT, median (IQR)	1:32 (1:32–1:64)	1:64 (1:32–1:128)	-
GMT (95% CI)	5.49 (5.25–5.73)	5.91 (5.69–6.12)	0.0103 0.36

CI: confidence interval, GMT: geometric mean titer, IQR: interquartile range, NAT: neutralizing antibody titer, RSV: respiratory syncytial virus. The GMT is shown in log_2_. * Paired *t*-test.

**Table 4:** Concentration of ESA at each NAT.

NAT (log_2_)	Site Ø			Site IIa		
	RSV-A (μg/mL) Mean ± SD	RSV-B (μg/mL) Mean ± SD	*p* *	RSV-A (μg/mL) Mean ± SD	RSV-B (μg/mL) Mean ± SD	*p* *
3	25.47 ± 10.29	35.44	-	30.03 ± 23.67	64.19	-
4	44.71 ± 20.02	33.30 ± 22.40	0.292	34.96 ± 25.34	29.22 ± 17.17	0.924
5	47.42 ± 24.52	49.67 ± 24.42	0.689	43.21 ± 23.85	35.89 ± 26.48	0.166
6	77.58 ± 35.19	53.84 ± 37.30	0.007	49.77 ± 28.63	37.20 ± 18.58	0.177
7	93.59 ± 36.86	65.67 ± 34.03	0.030	52.31 ± 31.21	45.36 ± 26.61	0.664
8	214.22 ± 133.73	113.02 ± 90.40	0.083	53.19 ± 24.78	57.15 ± 32.61	0.741
9	-	159.08 ± 145.11	-	-	65.42 ± 34.34	-

ESA: epitope-specific antibody, NAT: neutralizing antibody titer, RSV: respiratory syncytial virus. * Mann–Whitney test.

## Data Availability

Not applicable.
